# Sex-Specific and Strain-Dependent Effects of Early Life Adversity on Behavioral and Epigenetic Outcomes

**DOI:** 10.3389/fpsyt.2013.00078

**Published:** 2013-08-01

**Authors:** Marija Kundakovic, Sean Lim, Kathryn Gudsnuk, Frances A. Champagne

**Affiliations:** ^1^Department of Psychology, Columbia University, New York, NY, USA

**Keywords:** maternal separation, postnatal, brain, epigenetic, mice, strain differences, sex-dependent

## Abstract

Early life adversity can have a significant long-term impact with implications for the emergence of psychopathology. Disruption to mother-infant interactions is a form of early life adversity that may, in particular, have profound programing effects on the developing brain. However, despite converging evidence from human and animal studies, the precise mechanistic pathways underlying adversity-associated neurobehavioral changes have yet to be elucidated. One approach to the study of mechanism is exploration of epigenetic changes associated with early life experience. In the current study, we examined the effects of postnatal maternal separation (MS) in mice and assessed the behavioral, brain gene expression, and epigenetic effects of this manipulation in offspring. Importantly, we included two different mouse strains (C57BL/6J and Balb/cJ) and both male and female offspring to determine strain- and/or sex-associated differential response to MS. We found both strain-specific and sex-dependent effects of MS in early adolescent offspring on measures of open-field exploration, sucrose preference, and social behavior. Analyses of cortical and hippocampal mRNA levels of the glucocorticoid receptor (*Nr3c1*) and brain-derived neurotrophic factor (*Bdnf*) genes revealed decreased hippocampal *Bdnf* expression in maternally separated C57BL/6J females and increased cortical *Bdnf* expression in maternally separated male and female Balb/cJ offspring. Analyses of *Nr3c1*and *Bdnf* (IV and IX) CpG methylation indicated increased hippocampal *Nr3c1* methylation in maternally separated C57BL/6J males and increased hippocampal *Bdnf* IX methylation in male and female maternally separated Balb/c mice. Overall, though effect sizes were modest, these findings suggest a complex interaction between early life adversity, genetic background, and sex in the determination of neurobehavioral and epigenetic outcomes that may account for differential vulnerability to later-life disorder.

## Introduction

The experience of adversity in the early stages of development can have a profound impact on psychological and physical health. In humans, this phenomenon is illustrated in studies of prenatal exposure to stress and nutritional deprivation (1–4) as well as studies of postnatal neglect and abuse (5–7). Maternal exposure to famine during pregnancy has been found to predict increased risk of schizophrenia and antisocial personality disorder (8, 9) and a history of childhood neglect is associated with an increased risk of depressive disorders, drug abuse, and suicidality (6, 10). Importantly, these adverse experiences may not be deterministic in predicting later-life disorder, but instead generate a vulnerability to later-life stress or trauma. This model of disease etiology is perhaps best illustrated in the pathophysiology of post-traumatic stress disorder (PTSD). Risk of PTSD is significantly higher in individuals who have experienced early life stress (e.g., physical/sexual abuse, neglect) (11, 12) and individuals who experience early life stress are more likely to be exposed to trauma in later-life (13, 14). However, it is notable that only a relatively small percentage of individuals that experience early life trauma (approximately 25%) develop PTSD (15). Thus, understanding the factors that promote both risk and resilience to the effects of early life adversity is essential to further exploration of psychiatric dysfunction.

Though epidemiological and clinical studies have been informative regarding the consequences of exposure to prenatal and postnatal adversity, studies of the underlying biological mechanisms of these exposures have relied primarily on animal models. In primates and rodents, prolonged separations between mother and offspring have been used to model elements of childhood neglect/maltreatment and have provided experimental evidence for the emergence of neurobiological and behavioral abnormalities associated with this form of adversity (6). These studies have identified many changes, including altered hypothalamic-pituitary-adrenal (HPA) function (16, 17) and neuronal plasticity (18, 19), that are shaped by postnatal maternal separation (MS). More recently, epigenetic changes have been identified which may underlie these enduring physiological and neurobiological effects (20, 21). Epigenetic modifications, such as DNA methylation and post-translational histone modification, have been the increasing focus of efforts to determine the molecular pathways through which adversity becomes biologically embedded within the brain and other tissues (22). In humans, the experience of severe social deprivation (i.e., institutionalization from birth) or childhood abuse has been associated with altered DNA methylation profiles (23, 24). Psychiatric dysfunction is likewise linked to epigenetic variation in target genes and brain regions that have previously been implicated in the pathophysiology of these disorders (25–27). However, when considering the link between adversity, neurobiological dysfunction, and disorder, these human studies are limited by reliance on peripheral tissues (such as blood lymphocytes) or on post-mortem brain tissue, which may not necessarily map onto etiologically relevant epigenetic variation in the developing brain. Thus, animal models will continue to be critical methodological approaches in furthering our understanding of environmentally induced molecular and neurobiological change.

In the current study, our aim was to both determine the behavioral, brain gene expression, and DNA methylation changes induced by postnatal MS in mice and to determine whether these effects varied dependent on offspring strain and sex. There are a wide range of mouse strains/genotypes available for experimental laboratory studies and the “strain differences” in behavior of these mice have been well documented (28–32). Moreover, there is increasing evidence for the differential response of different strains of mice to environmental variation (33, 34). This differential responsiveness to environmentally induced behavioral change may also manifest in differential neurobiological and epigenetic change (35–38). Here we determined the effect of postnatal MS on C57BL/6J (B6) and Balb/cJ (Balb/c) mice – two strains with highly divergent behavioral phenotypes, particularly on measures of social/maternal, anxiety-like, and depressive-like behaviors (31, 35, 39, 40). In addition, within both strains, we determined the impact of MS on both male and female offspring. Sex-dependent effects of adversity have been shown in studies of prenatal stress (41, 42), *in utero* toxin exposure (43, 44), and postnatal maltreatment/neglect (45) and there is a significant sex-bias in the prevalence of most forms of psychopathology (46). Thus, it is of critical importance to understand the interaction between sex and exposure to adversity at a neurobiological and molecular level of analysis to determine the pathways through which these sex-dependent effects emerge. Moreover, there is increasing evidence that sex differences in themselves are associated with epigenetic variation – likely due to both genetic and hormonal differences between males and females (47, 48). Mother-infant interactions during postnatal development may likewise induce sex differences and have sex-dependent effects (49). Our experimental approach, through incorporation of both sex and strain was hypothesized to identify key variables that contribute to risk or resilience to adversity-induced effects.

## Results

**Figure 1:**
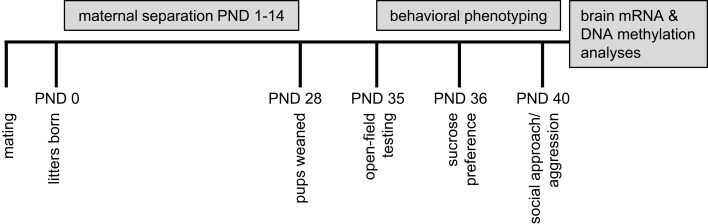
**Summary of experimental design**.

Study design is presented in Figure 1. The MS protocol (see Materials and Methods), involving prolonged, daily separation between dams and litters from postnatal days (PND) 1–14, was implemented in B6 and Balb/c mice and compared to a control rearing condition (standard laboratory rearing with no separation). From PND35 to PND 40, offspring were assessed on the following behavioral measures: open-field, sucrose preference, and social interaction. Following behavioral testing, in a subset of offspring, brains were dissected (prefrontal cortex and hippocampus) for analyses of gene expression and DNA methylation of the glucocorticoid receptor (*Nr3c1*) and brain-derived neurotrophic factor (*Bdnf*) genes. These gene targets were chosen as they have been previously demonstrated: (1) to be epigenetically regulated by DNA methylation (50, 51), (2) to exhibit plasticity in expression in response to a broad range of environmental exposures (23, 50, 52–54), and (3) to be within mechanistic pathways involved in HPA responsivity and neuroplasticity that have been implicated in the pathological psychiatric outcomes linked to the experience of adversity (55, 56).

### Maternal separation effects on open-field activity and exploration

**Figure 2:**
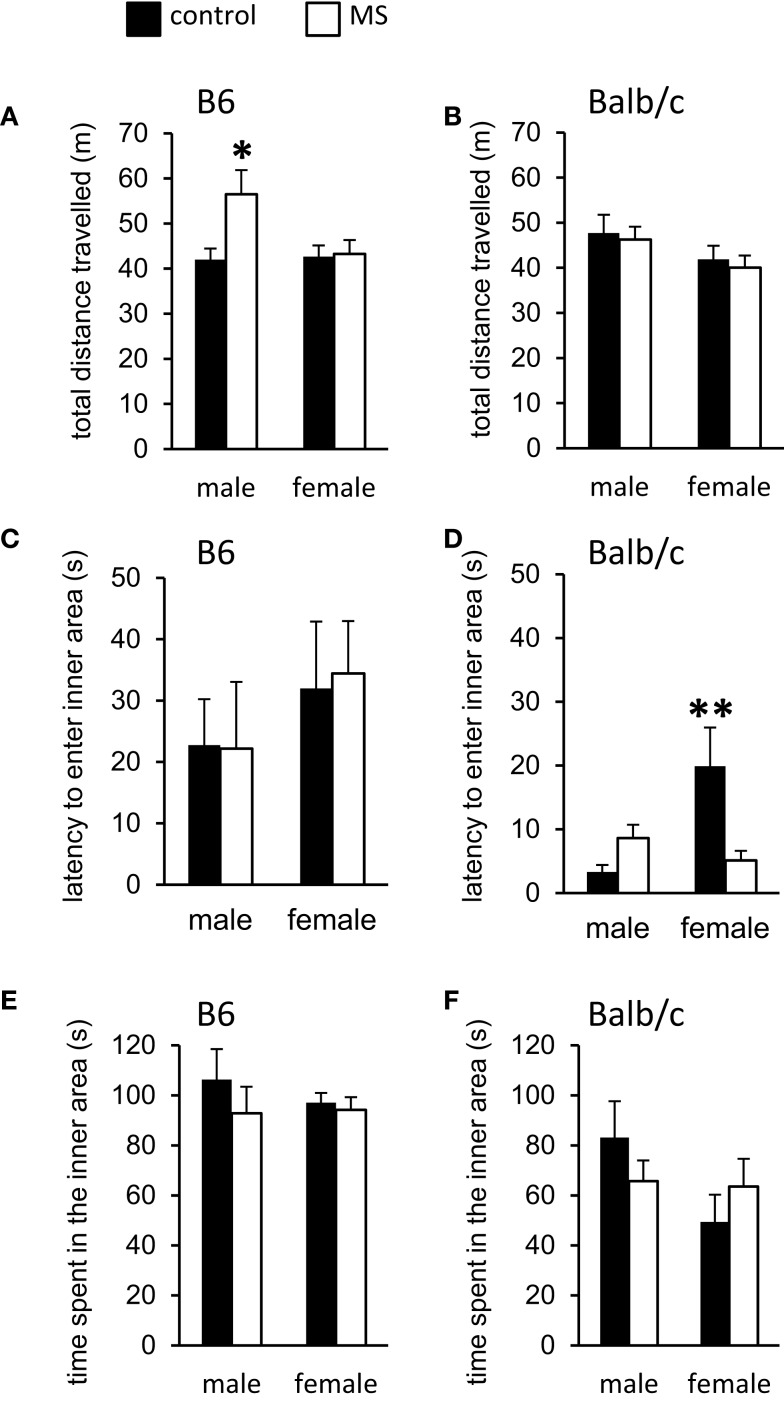
**Open-field behavioral effects of MS-rearing in B6 and Balb/c mice**. Open-field activity (total distance traveled) was **(A)** increased in MS-reared B6 males (*p* < 0.05) with **(B)** no effects in Balb/c mice. Latency to enter the inner area of the open-field was **(C)** not altered by MS in B6 mice and **(D)** was decreased in MS-reared Balb/c females (*p* < 0.01). No effects of MS were observed on time spent in the inner area of the open-field in **(E)** B6 or **(F)** Balb/c mice. *p < 0.05, **p < 0.01 (control vs. MS comparisons).

The open-field test is a standard measure of response to a novel environment (57). Activity (total distance traveled within the field) and exploration (movement within the anxiogenic inner area of the field) in rodents have been shown to differentiate individuals based on the experience of early life adversity (58, 59). In B6 mice, we found a rearing condition by sex interaction [*F*(1, 36) = 4.64, *p* < 0.05] on total distance traveled during testing, such that MS-reared males exhibited increased activity levels compared to control-reared males, with no rearing effect in B6 females (Figure 2A). In contrast, MS had no effect on activity levels in Balb/c mice (Figure 2B). Latency to enter the inner/anxiogenic area of the open-field was not found to be altered by rearing condition in B6 mice (Figure 2C). In Balb/c mice, we found a significant sex-specific rearing condition effect on this measure, with MS-reared females exhibiting shorter latencies to enter the inner area compared to control-reared females [χ^2^(1, 19) = 8.13, *p* < 0.01; Figure 2D]. Time spent in the inner area of the open-field, a typical measure of anxiety-like behavior (57), was not found to be altered by rearing condition in B6 or Balb/c mice (Figures 2E,F).

### Maternal separation effects on sucrose preference

Preference for sucrose vs. water is used as a measure of reward sensitivity or hedonic motivation and in animal models of depression, a reduction in preference for sucrose is typically observed (60–62). Consistent with previous reports (63), we found Balb/c mice to have overall reduced sucrose preference compared to B6 mice. All mice exhibited a higher than 50% average sucrose consumption (range 53–95%), indicating that the sucrose solution used was sufficiently rewarding and that no aversion to the sucrose solution was observed. We classified mice as having a preference for sucrose if they consumed more than 75% sucrose (as a percentage of total consumption) across the 3-day testing period. This definition of “preference” is consistent with previous studies of motivation in which the preferred stimulus must be favored 25% more than the comparison stimulus (64). Within B6 mice, both males and females that had experienced MS displayed reduced sucrose preference [males: χ^2^(1, 18) = 2.38, *p* < 0.05; females: χ^2^(1, 19) = 2.22, *p* < 0.05; Table 1]. Interestingly, within Balb/c mice, we observed sexual dimorphism in sucrose preference in control animals (males consumed more sucrose than females) that was reversed by MS; MS-reared males exhibited reduced sucrose preference whereas MS-reared females exhibited elevated sucrose preference [males: χ^2^(1, 19) = 2.45, *p* < 0.05; females: χ^2^(1, 19) = 2.78, *p* < 0.05; Table 1].

**Table 1:** **Percentage of mice exhibiting sucrose preference**.

		Control %	MS %
B6	Male	63	43*
	Female	56	28*
Balb/c	Male	50	20*
	Female	30	60*

Statistically significant MS-induced effects are indicated in bold font; *p < 0.05 control vs. MS.

### Maternal separation effects on social approach and aggression

**Figure 3:**
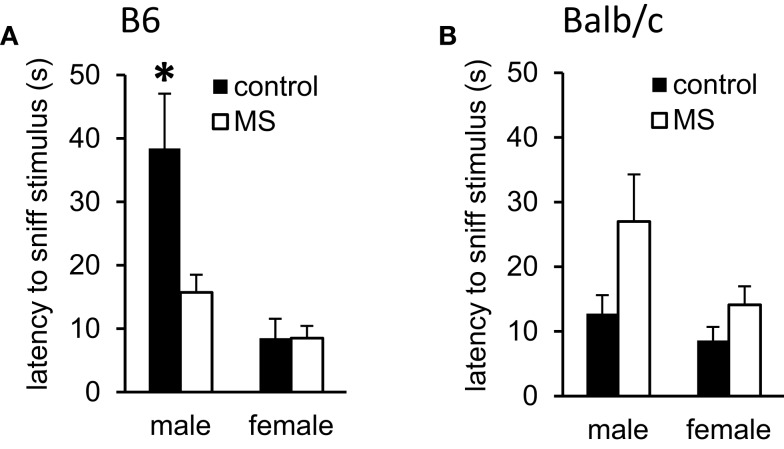
**Effects of MS-rearing on social behavior**. Latency to sniff a novel mouse during dyadic social interactions was **(A)** decreased in MS-reared B6 males (*p* < 0.01) with no effect observed on this measure in **(B)** Balb/c mice. *p < 0.05 (control vs. MS comparisons).

Deficits in social behavior are a core feature in many forms of psychopathology (65) and impaired social interactions have been observed following exposure to reduced mother-infant interactions (66). Latency to sniff and aggressive behavior during dyadic social encounters with a novel stimulus mouse (129Sv strain) were assessed in control-reared vs. MS-reared mice. In B6 mice, we found a sex-specific rearing condition effect on latency to sniff the stimulus mouse, with shorter latencies observed amongst MS-reared B6 males [χ^2^(1, 16) = 7.61, *p* < 0.05] and no effect of rearing condition in B6 females (Figure 3A). No rearing condition effects were observed in Balb/c mice (Figure 3B). Across strains, aggressive behavior was only observed in males. Likelihood of displaying aggressive behavior was significantly increased in MS-reared Balb/c males (control: 66.7% vs. MS: 90%, *p* < 0.05) while this effect was not observed in B6 males (control: 30.5% vs. MS: 42.9%).

### Effect of maternal separation on cortical and hippocampal gene expression

Within the prefrontal cortex and hippocampus, we analyzed relative mRNA levels of *Nr3c1* and *Bdnf*. In B6 mice, MS was generally associated with a decrease in *Nr3c1* and *Bdnf*, though this effect was only statistically significant for *Bdnf* mRNA levels within the hippocampus (Table 2). Here we found a significant rearing condition by sex interaction [*F*(1, 23) = 3.90, *p* < 0.05], where B6 females that experienced MS had decreased *Bdnf* mRNA, with no rearing effect in males. In Balb/c mice, we found increased *Bdnf* mRNA in the prefrontal cortex of MS mice [both sexes; *F*(1, 23) = 8.05, *p* < 0.01; Table 2]. No other gene expression changes were noted in this mouse strain.

**Table 2:** **Relative mRNA levels of *Nr3c1* and *Bdnf* in the prefrontal cortex (PFC) and hippocampus (HIPP)**.

			*Nr3c1*		*Bdnf*	
			Control	MS	Control	MS
B6	PFC	Male	1.01 ± 0.06	0.84 ± 0.05	1.05 ± 0.12	1.00 ± 0.21
		Female	1.02 ± 0.11	0.98 ± 0.09	1.10 ± 0.12	0.86 ± 0.11
	HIPP	Male	1.02 ± 0.10	0.93 ± 0.06	1.04 ± 0.11	1.06 ± 0.13
		Female	0.96 ± 0.09	0.83 ± 0.09	1.03 ± 0.04	0.66 ± 0.07*
Balb/c	PFC	Male	1.01 ± 0.07	0.92 ± 0.08	1.04 ± 0.12	1.29 ± 0.10**
		Female	1.01 ± 0.07	1.05 ± 0.10	0.96 ± 0.09	1.30 ± 0.08**
	HIPP	Male	1.02 ± 0.08	1.03 ± 0.05	1.04 ± 0.10	1.32 ± 0.05
		Female	1.01 ± 0.10	0.83 ± 0.06	1.00 ± 0.09	1.00 ± 0.14

Statistically significant MS-induced effects are indicated in bold font; *p < 0.05, **p < 0.01 (control vs. MS comparisons).

### DNA methylation changes associated with maternal separation

**Figure 4:**
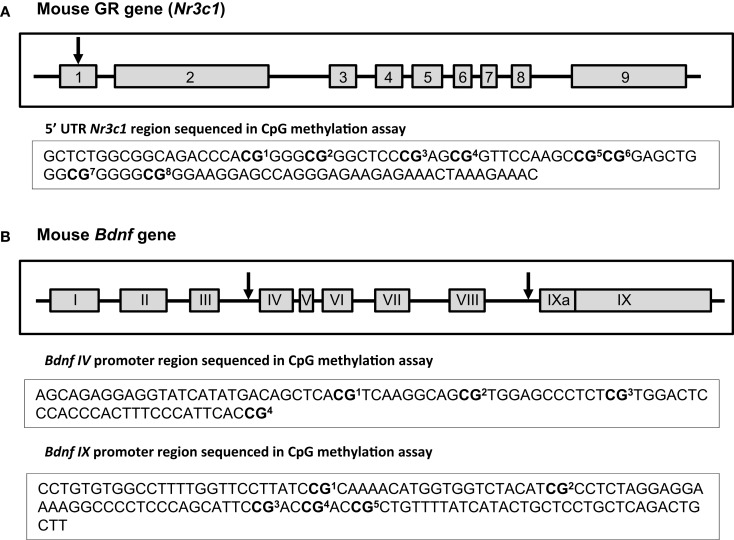
**Schematic of *Nr3c1* and *Bdnf* genes**. Exons are depicted as gray boxes and the introns as lines. Numbers of *Nr3c1* exons **(A)** are indicated in Arabic numerals while numbers of *Bdnf* exons **(B)** are indicated in Roman numerals to conform to standard nomenclature. The arrows show the approximate location of the examined sites within those genes. The sequences under each scheme show the exact CpG sites that were analyzed in 5′UTR region of *Nr3c1* **(A)** and in *Bdnf* promoter regions IV and IX **(B)** using bisulfite-pyrosequencing method [the schemes of *Nr3c1* and *Bdnf* genes were adapted from (67) and (68) respectively].

**Figure 5:**
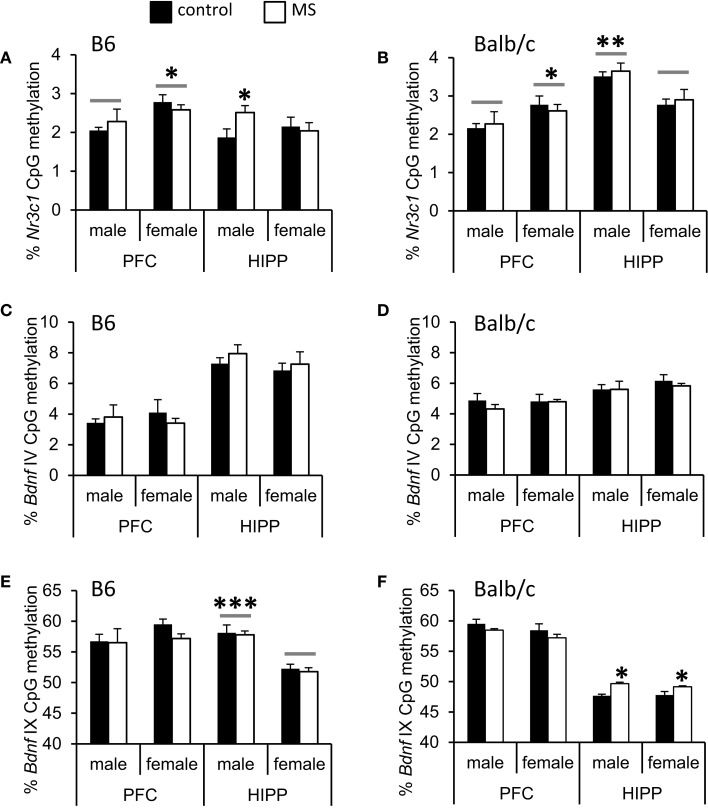
**Average percent DNA methylation of the *Nr3c1* and *Bdnf* promoter regions in the cortex (PFC) and hippocampus (HIPP)**. **(A)** Increased *Nr3c1* DNA methylation was observed in the HIPP of MS-reared B6 males and **(B)** no MS-rearing effects on DNA methylation of this gene in Balb/c mice. In the PFC, sex differences (indicated by a gray bar) were present in both B6 and Balb/c mice (elevated *Nr3c1* DNA methylation in females compared to males). In the hippocampus, Balb/c females had reduced *Nr3c1* DNA methylation compared to males. *Bdnf IV* promoter DNA methylation was not altered by MS-rearing in **(C)** B6 or **(D)** Balb/c mice. MS-rearing had **(E)** no effect on *Bdnf IX* promoter DNA methylation in B6 mice but **(F)** increased DNA methylation of this region in the HIPP of Balb/c mice. In B6 mice, females had reduced *Bdnf IX* promoter DNA methylation in the hippocampus compared to males (indicated by gray bars). *p < 0.05, **p < 0.01, ***p < 0.001 (control vs. MS comparisons or male vs. female comparisons).

We analyzed DNA methylation across 8 CpG sites within the *Nr3c1* promoter region (see Figure 4A), which is highly homologous to the rat exon 1_7_ GR promoter (50); this region also contains the binding site for the transcription factor NGFI-A (CpGs 7 and 8; Figure 4A). Analyses were conducted on average levels of DNA methylation across the 8 CpG sites to reduce multiple testing. In B6 mice, we found a significant rearing condition by sex interaction [*F*(1, 23) = 3.85, *p* < 0.05; Figure 5A], with elevated hippocampal CpG methylation in MS-reared males and no rearing effects in females. No rearing effects on GR methylation were detected in Balb/c mice (Figure 5B) or in the prefrontal cortex of B6 mice (Figure 5A). Within both strains, we found differences in CpG methylation associated with sex, such that in the prefrontal cortex there were elevated levels of methylation in females compared to males [B6: *F*(1, 23) = 6.90, *p* < 0.05; Balb/c: *F*(1, 23) = 5.08, *p* < 0.05]. Within the hippocampus, the converse was evident in Balb/c mice, with males having elevated DNA methylation levels compared to females [*F*(1, 23) = 14.74, *p* < 0.01; Figure 5].

We examined DNA methylation status of two regions of the *Bdnf* gene known to be epigenetically regulated: promoter region IV (51, 69) and promoter region IX (70) (see Figure 4B). Within the *Bdnf* IV promoter region, we analyzed DNA methylation across four CpG sites, including the CpG that lies within the binding site of the transcription factor CREB (CpG 1; Figure 4B). As with *Nr3c1*, analyses were conducted on average levels of DNA methylation across *Bdnf* CpG sites to reduce multiple testing. We found no rearing effects on DNA methylation (Figures 5C,D). Within the *Bdnf* IX promoter region (see Figure 4B), we analyzed DNA methylation across 5 CpGs. We found a rearing condition effect in the hippocampus of Balb/c mice, with increased DNA methylation associated with MS (both sexes) [*F*(1, 23) = 4.82, *p* < 0.05; Figure 5F]. No other rearing effects were determined (Figures 5E,F). Within promoter IX of the *Bdnf* gene, B6 males were found to have elevated hippocampal DNA methylation compared to females [*F*(1, 23) = 51.43, *p* < 0.001].

## Discussion

Our findings support the hypothesis that MS induces changes in behavior, brain gene expression, and DNA methylation in inbred mice. These findings also provide evidence for strain differences in response to MS and the interaction between sex and rearing experience in the prediction of these outcome measures. It does not appear to be the case that there is an overall “differential susceptibility” amongst B6 vs. Balb/c mice in their responsiveness to MS as there is evidence for MS-induced effects in both strains. However, strain responsiveness to MS does vary between measures, resulting in rearing effects in B6 mice on measures of open-field activity, sucrose preference, latency to approach a novel social stimulus, hippocampal *Bdnf* mRNA levels, and hippocampal *Nr3c1* DNA methylation. In contrast, rearing effects in Balb/c mice were observed on latency to enter the inner area of the open-field, sucrose preference, aggressive behavior toward a novel stimulus mouse, *Bdnf* mRNA levels in the prefrontal cortex, and DNA methylation of the *Bdnf IX* promoter region in the hippocampus. Even within the one measure that is altered in both mouse strains as a function of rearing environment – sucrose preference – the within-strain effect is different, with B6 males and females both exhibiting reduced sucrose preference and an interaction between sex and rearing condition in Balb/c mice (males showing decreased and females showing increased preference). Overall, these findings suggest that adversity experienced during postnatal development can manifest in divergent effects dependent on broad genetic characteristics, such as strain, and dependent on the sex of the individual experiencing the adversity; findings which point toward a very complex interplay between these individual- and group-level characteristics, the environment, and risk phenotypes.

### Epigenetic effects of adverse environments

Though investigation of the effects of MS on behavioral and neurobiological outcomes is well established within the literature (16, 17), the incorporation of epigenetic analyses within these experimental designs is a relatively recent approach. In mice, MS-rearing has been previously demonstrated to induce hypomethylation of the vasopressin gene (*Avp*) within the hypothalamus leading to increased HPA reactivity amongst MS-reared offspring (20). Exposure to a single 24-h MS at PND9 has been associated with increased *Avp* DNA methylation in B6 mice and increased *Nr3c1* DNA methylation in DBA/2J mice (38). Similar to our findings, this study highlights the divergent epigenetic effects of MS in different mouse strains. Increased DNA methylation within the *Mecp2* (methyl CpG binding protein 2) and cannabinoid receptor-1 genes and decreased DNA methylation within the corticotropin releasing factor receptor 2 (*Crfr2*) gene has also been observed in the cortex of MS-reared B6 mice (21). Interestingly, these epigenetic changes were also observed in the sperm of MS-reared males and may account for the transmission of behavioral and epigenetic effects of MS-rearing across generations (21, 71). Beyond DNA methylation, there is also evidence for post-translational modification to histones associated with MS-rearing and pharmacological inhibition of histone deacetylases prior to MS can prevent the emergence of MS-associated risk phenotypes (72–74). Comparison of B6 and Balb/c mice on MS-induced histone changes suggests that altered cortical histone deacetylase mRNA (increased in juveniles and decreased in adults) is associated with MS-rearing in Balb/c but not B6 mice and that these enzymatic changes are associated with age-dependent differences in histone (H4) acetylation (73). This study suggests a biphasic epigenetic response to adversity that may have consequences for the developmental timing of phenotypic (physiological, neurobiological, behavioral) outcomes associated with MS.

The epigenetic effects of MS contribute to a growing literature on the adverse effects of a broad range of early life experiences. In rodents, prenatal stress (42), nutrient deprivation (75, 76), variation in maternal care (50, 77), postnatal abuse (52), and post-weaning social environments (78) have been observed to induce epigenetic effects (DNA methylation and/or histone modifications). The *Nr3c1* and *Bdnf* genes examined in the current study appear to be highly plastic in expression and epigenetic regulation in response to these experiences (50, 52). These gene targets are also linked to the neurobiological pathways which may underlie risk of psychopathology. Glucocorticoid receptors within the hippocampus serve a critical negative-feedback role within the HPA axis such that elevated levels of these receptors are associated with an increased capacity to down-regulate the stress response and return to baseline glucocorticoid levels (55). Adverse early life experiences are typically associated with decreased *Nr3c1* expression levels and increased DNA methylation of the promoter region of this gene (23, 50). Though we did not find significant reductions in hippocampal *Nr3c1* expression, DNA methylation within the *Nr3c1* promoter was increased in MS-reared B6 males. *Bdnf* confers neuronal plasticity and has been demonstrated to alter mood and cognition (56, 79). Adverse early life experiences, such as abuse, have been demonstrated to decrease *Bdnf* expression and increase *Bdnf* DNA methylation (52). Our data are consistent with this previous research, though it is notable that we observed decreased *Bdnf* expression in hippocampal tissue of B6 MS-reared females whereas increased *Bdnf* DNA methylation was only observed in MS-reared Balb/c mice. Intriguingly, we found increased *Bdnf* expression in the prefrontal cortex of MS-reared Balb/c mice that was not associated with changes in DNA methylation of the examined CpG sites. The lack of correspondence between expression and DNA methylation highlights the complex regulatory networks that may be recruited by MS-rearing and may vary over time. For instance, although DNA methylation changes have the potential to induce long-lasting changes in gene expression (50), it is possible that compensatory mechanisms may override the effect of DNA methylation on gene regulation. In addition, the behavioral testing of these individuals, which may alter gene expression and DNA methylation independent of rearing condition, may have limited our ability to provide a clear correlation between MS-induced DNA methylation and gene expression. However, it is important to acknowledge that DNA methylation is only one of many epigenetic mechanisms that can regulate gene expression and so it may be the case that variation in DNA methylation is not causally related to the gene expression changes we observed in the current study.

It is also worth noting the limitations of our gene expression/epigenetic analyses. First, we examined only total *Bdnf* mRNA levels and it is possible that changes in specific (particularly low-abundance) *Bdnf* transcripts were not detected due to a dilution effect. In addition, we examined only DNA methylation of the CpG sites in the *Bdnf* promoter regions IV and IX, previously shown to be epigenetically regulated (51, 69, 70). Thus, it is possible that MS could have induced epigenetic changes in *Bdnf* promoter regions not examined in this study. There is increasing evidence for epigenetic variation at CpG shores rather than promoter CpG islands (80) and so loci outside of the regions analyzed might be more relevant to MS-induced effects. Finally, the DNA methylation changes we observed were modest and it is difficult to evaluate the biological relevance of changes of this magnitude derived from the current methodological approaches used for *in vivo* analyses. It seems likely that MS-induced epigenetic effects are specific to a sub-population of cells within the brain regions examined and thus are diluted through the inclusion of multiple neuronal and glial cells. Therefore, future studies of MS-induced epigenetic changes would benefit from cell-type specific analyses that may facilitate our efforts to detect epigenetic and gene expression changes that are induced by early life adversity and contribute to behavioral abnormalities occurring later in life.

The rapid development of methodologies for assessing epigenetic variation has also provided opportunities to determine the translational relevance of research on adversity-induced changes in DNA methylation. In post-mortem brain tissue, increased hippocampal DNA methylation of the *Nr3c1* promoter and decreased *Nr3c1* expression is observed in individuals with a history of childhood abuse (23). Similar adversity-associated increases in *Nr3c1* promoter methylation have been documented in humans in non-neuronal tissues such as fetal cord blood (81, 82), blood lymphocytes (83), and buccal cells (84). Genome-wide DNA methylation analyses of blood lymphocytes suggest that global DNA hypermethylation may result from childhood social/maternal deprivation (being reared in an institution vs. reared by biological parents) (24). The question raised by these intriguing findings is the relevance of peripheral epigenetic markers for predicting epigenetic variation in the brain – particularly in light of the goal to further our understanding of the neurobiological pathways through which adversity leads to psychopathology. We have previously found limited concordance between peripheral and brain tissues in DNA methylation levels of the *Nr3c1* gene promoter (67). Within the current study, though peripheral tissues were not assessed, it is clear that MS has a unique epigenetic impact in different brain regions (i.e., *Nr3c1* and *Bdnf* MS-associated DNA methylation changes observed in the hippocampus and not the prefrontal cortex). Thus, even within the brain, epigenetic responsiveness may not be consistent across genes. This observation does not invalidate approaches using peripheral tissue to predict neuronal changes but does suggest that the complexity of tissue-specific molecular responses and the mechanisms through which both peripheral and brain tissues would be affected by adverse environmental experiences need to be carefully considered.

### Sex-specific outcomes associated with adversity

Sex differences in response to early life experiences are a relatively consistent finding within the literature. In humans, childhood maltreatment may increase rates of depression and drug use in females, with more limited effects in males (85). On neuroendocrine measures, sex is a significant modulator of the relationship between childhood adversity and HPA activity (86). This sex-specificity is also observed following prenatal adversity. Exposure to *in utero* stress/nutrient deficiency during pregnancy may increase the risk of schizophrenia in males but not females (87) and maternal bereavement stress during pregnancy has been found to increase the risk of attention deficit disorder in males (88). However, these effects may be due in part to the sex-bias in these disorders induced by hormonal and genetic differences (with males having higher rates than females) (89). Animal studies likewise suggest the sex-specificity of early life adversity (42) and in the current study, sex by rearing condition interactions are the norm rather than the exception. Similar to the effect of strain, our findings support the hypothesis that both males and females are sensitive to the effects of MS, but that the effects of MS manifest in different ways dependent on sex. We have found that B6 males, but not B6 females, exhibit hyperactivity in response to MS. Similarly, Balb/C males are more vulnerable to MS-induced anhedonia than Balb/C females. In addition, we have recently shown that the differential response of males and females to early life toxicological exposures can be observed at the level of gene expression and DNA methylation in the brain corresponding to changes in social and anxiety-like behavior (44). Sex-specific epigenetic effects are an emerging theme in the study of early life adversity and may account for the sex-bias in adversity-associated behavioral and neurobiological dysfunction. Interestingly, in the current study we observed sex-specific gene expression and epigenetic variation in B6 mice, whereas in Balb/c mice, male and female effects of MS-rearing are similar. Consistent with previous reports (54), we also find sex differences in DNA methylation regardless of rearing condition. These findings add another layer of complexity, which includes differential genetic background, to the investigation of sex-specific responsiveness to adversity.

### Can adversity lead to improved outcomes?

Though the experience of disruption to the *in utero* environment or childhood maltreatment is linked to psychiatric dysfunction (7, 8), it is clear that there is a significant degree of resilience to early life adversity (15, 90). Within the current study, the effects of MS-rearing are relatively modest suggesting that, similar to human populations, many individuals are resilient to MS. However, in addition to these indices of resilience, we find that MS-reared Balb/c females will more rapidly enter the anxiogenic center area of the open-field and have increased sucrose preference. These behavioral phenotypes would suggest reduced anxiety- and depressive-like phenotypes as a function of early life adversity. In light of these perplexing findings, one hypothesis is that adversity can lead to improved outcomes dependent on sex and genetic background. Several lines of evidence may be relevant to evaluating the plausibility of this hypothesis. In primates, early, intermittent periods of MS have been documented to reduce indices of anxiety-like behavior and enhance HPA negative-feedback, suggesting a protective effect of early life adversity (91–93). There is also evidence for enhancements in functioning following exposure to adverse experiences, if the adversity is constant across developmental periods. Though maternal depression during pregnancy can predict impairments in functioning, there is enhanced motor and neuronal development in infants that experienced maternal depression during both *in utero* and postnatal periods (compared to infants who were only exposed to maternal depression at one developmental timepoint) (94). Previous studies of Balb/c mice have shown that the *in utero* environment of this mouse strain can exert significant programing effects, leading to increased anxiety-like behavior (95). It may be the case that MS during postnatal development in this strain generates a better environmental “match” to the prenatal environment, allowing the neuroendocrine adaptations of offspring to enhance functioning. Though these are hypotheses that have yet to be tested, the phenomenon of improved functioning following adversity in a subset of individuals should not be dismissed.

### Inter-individual variability in the effects of maternal separation

The relatively modest effects of MS-rearing that we observe in the current study and the inconsistent effects of MS observed in previous studies (17) requires careful examination of the MS paradigm and the hypothesized pathways through which this form of adversity alters offspring development. Prolonged separations between mothers and offspring are thought to model childhood neglect and the stress of this manipulation has been found to reduce mother-infant interactions during the post-reunion period (96). However, these group-level effects may not be observed in all litters and certainly there are individual differences in the frequency of mother-infant interactions under standard rearing conditions that have significant programing effects on brain and behavior (97, 98). These individual differences in maternal behavior likely contribute to the variability in response to adversity. The use of MS combined with maternal stress during the separation period is one approach intended to create a more consistent reduction in maternal behavior in MS litters and this methodology has previously been found to reduce mother-infant interactions in mice (21). However, this approach does not account for the variability in maternal care in control litters and does make the interpretation of the role of MS vs. maternal care on outcome measures problematic. This will be an important issue to address in subsequent studies using the current MS protocol.

A second issue to consider within the MS paradigm is how the individual responsiveness to adversity may be used to better understand the molecular and neurobiological basis of risk and resilience. In the current study, we examined gene expression and DNA methylation in a random subset of individuals. However, perhaps a more powerful strategy for assessing the link between adversity, neurobiological changes, and risk phenotypes would be to stratify the sample with comparisons between those individuals that manifest risk phenotypes (increased anxiety- and depressive-like behavior) and those individuals that are resilient. Within the context of studies aimed at understanding the etiological pathways leading to psychopathology, this approach, combined with a more detailed assessment of the characteristics of the postnatal environment, may provide a more informative experimental paradigm that can advance our understanding of the biological basis of adversity-induced dysfunction.

### Future directions

The strain and sex-dependent effects of MS that we identified in the current study highlight the complexity of the effects of early life adversity. Though strain and sex differences in neurobiology and behavior are well documented, the molecular basis of the differential response to environmental exposures has yet to be elucidated. Epigenetic analyses within future studies of these effects may advance our understanding of this differential response and should be combined with experimental designs where important modulating variables, such as prenatal and postnatal maternal effects, are assessed. Within-individuals, the differential epigenetic response of different tissues (brain and peripheral) over multiple timepoints may provide important insights into the pathways leading to risk phenotypes and contribute to translational studies of the impact of early life adversity.

## Materials and Methods

### Animals

C57BL/6J (B6) and Balb/cJ (Balb/c) mice (Jackson Laboratories) were used in these studies. Adult males (*n* = 10) and females (*n* = 20) of each strain were housed two per cage in 10.5″ × 19″ × 6″ cages and habituated to the animal facility in the Department of Psychology at Columbia University for 2 weeks prior to mating. At mating, two females were housed with one male for 10 days. This mating protocol generated *n* = 13 B6 and *n* = 14 Balb/c litters. At birth (PND0), all pups were counted and weighed. Animals were maintained at a constant temperature and humidity with a 12L:12D light schedule (lights off 10:00 a.m.) and *ad libitum* access to chow and water. All procedures were performed in accordance with guidelines of the NIH regarding the Guide for the Care and Use of Laboratory Animals and with the approval of the Institutional Animal Care and Use Committee (IACUC) at Columbia University.

### Postnatal maternal separation

Starting on PND1, litters were exposed to daily MS or standard laboratory rearing conditions (see Figure 1). The protocol, previously used in (21), involved 2 h of daily separation of pups and dam from PND1 to PND14 combined with maternal exposure to unpredictable stress during the period of separation. At the start of the separation period, dams were removed from the home-cage and placed in a clean cage with *ad libitum* access to chow and water. Pups were also removed from the home-cage and placed together in a clean cage. At a randomly selected time within the 2-h separation, dams were exposed to 20 min of restraint stress or 2 min of forced swim. During restraint, females were removed from the temporary housing cage and placed in a conical tube that restricted all vertical and horizontal movement. During forced swim, mice were placed in a 2 l glass beaker containing 1 l of water (20°C). After the 2-min period, mice were patted dry with a towel and returned to the temporary housing cage.

### Reproductive outcomes

The breeding protocol used in the current study resulted in a 65 and 70% rate of successful births in Balb/c and B6 mice, respectively. Average litter weights at PND0 and PND6, litter size at PN6, litter mortality rates during the first postnatal week, litter sex ratio, and average weaning weights of male and female offspring are provided in Table 3. No significant rearing condition effects were observed except on the measure of male pup weaning weights, which were decreased in MS-reared Balb/c males compared to control-reared Balb/c males [*t*(1, 12) = 3.03, *p* < 0.05]. Litters containing fewer than two pups at the time of weaning (PND28) were excluded, resulting in *n* = 6 litters per strain for the control rearing condition and *n* = 8 B6 and *n* = 7 Balb/c litters for the MS-rearing condition. For behavioral measures, one to two pups per sex per litter were tested (*B6*: control male, *n* = 10; control female, *n* = 9; MS male, *n* = 7; MS female, *n* = 11; *Balb/c*: *n* = 10/sex/rearing condition). For these analyses, litter was used as a covariate. For gene expression and DNA methylation analyses, only one pup (per sex) was used per litter with a sample size of *n* = 6 pups per sex per rearing condition.

**Table 3:** **Reproductive outcomes (mean ± SEM) in control and MS litters**.

		Av. birth weight	PN6 litter size	PN6 pup av. weight	Litter sex ratio (m/f)	% Pup mortality^1^	Av. weaning weight (m)	Av. weaning weight (f)
B6	Control	1.27 ± 0.03	6.00 ± 0.82	3.04 ± 0.40	1.11 ± 0.70	13.65 ± 4.67	15.85 ± 1.08	13.47 ± 0.27
	MS	1.32 ± 0.05	5.14 ± 0.83	3.61 ± 0.25	0.90 ± 0.86	22.02 ± 8.94	15.55 ± 0.61	17.85 ± 4.38
Balb/c	Control	1.36 ± 0.06	6.00 ± 0.63	3.55 ± 0.50	1.22 ± 0.65	13.16 ± 6.41	**15.40 ± 0.44**	14.20 ± 0.92
	MS	1.41 ± 0.04	5.75 ± 0.65	3.68 ± 0.30	1.01 ± 0.89	5.90 ± 3.87	13.36 ± 0.50*	13.16 ± 0.50

Statistically significant MS-induced effects are indicated in bold font; *p < 0.05 control vs. MS; ^1^mortality occurring between PN0 and PN6 (no mortality was observed after this period).

#### Behavioral assessment

At PND28, all offspring were weaned and commenced behavioral testing at PND35 (see Figure 1). All offspring underwent testing in the open-field apparatus (PND35), assessed for sucrose preference (PND36–39), and then observed during a dyadic social encounter with a stimulus mouse in the open-field apparatus (PND40). Testing during juvenile/adolescent development was conducted to determine the emergence of behavioral risk phenotypes at this early period, prior to the onset of full sexual maturity, and create further parallels with studies in humans that have observed childhood and adolescent behavioral problems that are predicted by adversity and predictors of later-life risk of psychopathology (99–101). However, it should be noted these behavioral tests have been validated in adult rather than juvenile/adolescent mice.

### Open-field testing

The open-field apparatus used was a 24″ × 24″ × 16″ black plastic box. On the day of testing, the mouse was placed directly into one corner of the open-field. After a 10-min session, the mouse was returned to its home-cage. All testing was conducted under red lighting conditions and tests were video recorded. Behaviors scored using Ethovision (Noldus) included: (1) distance traveled, (2) latency to enter the center area, and (3) center area exploration (time spent in the inner 12″ × 12″area).

### Sucrose preference

Immediately following open-field testing, mice were singly housed and placed in a cage with two water bottles (both containing water). The following day, on PND36, both bottles were removed. One bottle was filled with water, weighed, and placed in the cage. The second bottle was filled with a 1% sucrose solution, weighed, and placed in the cage. Each day, bottles were weighed to determine consumption levels (three consecutive days). The position of the sucrose vs. water bottle was alternated each day to avoid place preference. Sucrose preference was defined as having average sucrose consumption levels (averaged across the 3-day period) of 75% or higher. Percentage consumption levels were defined as total sucrose consumed divided by the total volume of liquid consumed (water + sucrose). Sucrose preference was stable over consecutive days in both control and MS mice suggesting that initial reactivity to single housing (conducted on the day prior to sucrose preference testing) did not contribute to the rearing condition effects observed.

### Social behavior

At PND40, a subject mouse was placed in the open-field apparatus with a same-sex stimulus mouse (129Sv) for 30 min. Sessions were video recorded. Latency to sniff/approach the stimulus and occurrence of aggressive behaviors (tail rattling, chasing, biting) were coded.

### Nucleic acid isolation

Following assessment of social behavior at PND40, mice were sacrificed by rapid decapitation and brains extracted and stored at −80°C. Whole hippocampus and cortical tissue containing the prefrontal cortex were dissected from partially thawed tissue and Allprep DNA/RNA mini kit (Qiagen) was used for simultaneous extraction of total RNA and genomic DNA.

### Quantitative real-time PCR

Gene expression was assessed using reverse transcription (The SuperScript^®^ III First-Strand Synthesis System, Invitrogen) followed by quantitative real-time PCR with a 7500 real-time PCR system (Applied Biosystems). Using specific primer sets (see Table 4), mRNA levels of the glucocorticoid receptor (*Nr3c1*) and brain-derived neurotrophic factor (*Bdnf*) were determined. Relative mRNA expression was calculated using the standard ΔΔCT method (102) with male control samples as a reference sample and cyclophilin A (*CypA*) and beta-actin (*Actb*) as endogenous reference genes.

**Table 4:** **Primers for gene expression analyses**.

Gene name	Forward primer	Reverse primer
*Nr3c1*	AACTGGAATAGGTGCCAAGG	GAGGAGAACTCACATCTGGT
*Bdnf*	CATAAGGACGCGGACTTGTACA	AGACATGTTTGCGGCATCCA
*CypA*	GAGCTGTTTGCAGACAAAGTTC	CCCTGGCACATGAATCCTGG
*Actb*	TATTGGCAACGAGCGGTTCC	TGGCATAGAGGTCTTTACGGATGTC

### Bisulfite-pyrosequencing

DNA methylation at specific CpG sites in the *Nr3c1* and *Bdnf* genes was analyzed using bisulfite-pyrosequencing method. Bisulfite conversion of DNA samples (500 ng) was carried out using EpiTect Bisulfite Kit (Qiagen). Biotinylated PCR products were obtained using PyroMark PCR kit (Qiagen) and PCR primers specific for *Nr3c1* and *Bdnf* gene regions (see Figure 4). Pyrosequencing was performed on a PyroMark Q24 Pyrosequencer using specific pyrosequencing primers (see Table 5). Average DNA methylation levels of CpG sites were quantified using PyroMark Q24 2.0.4. Software (Qiagen).

**Table 5:** **PCR and pyrosequencing primers used for DNA methylation analysis**.

MOUSE GR GENE (*Nr3c1*) – chr18:39,649,906-39,650,025*	
PCR primer – forward	GGTTTTGTAGGTTGGTTGTTATTT
PCR primer – reverse – Biotinylated	/5Biosg/TCTCTTCTCCCTAACTCCTT
Pyrosequencing primer	GGGTTTTGGAGGTAGATTTA
MOUSE BDNF PROMOTER IV (*Bdnf IV*) – SITES IV1-IV4 – chr2:109,532,399-109,532,715*	
PCR primer – forward	TAGGATTGGAAGTGAAAATATTTATAAAGT
PCR primer – reverse – Biotinylated	/5Biosg/CCTTCAACCAAAAACTCCATTTAATCT
Pyrosequencing primer	AGAGGAGGTATTATATGATAG
MOUSE BDNF PROMOTER IX (*Bdnf IX*) – SITES IX5-IX1 – chr2:109,562,918-109,563,064*	
PCR primer – forward	GGTGTTTGGTGTTTTAAGTAGTT
PCR primer – reverse – Biotinylated	/5Biosg/ACAAATCCTATATAACCTTTTAATTCC
Pyrosequencing primer	TGAGTAGGAGTAGTATGATAA

*Genomic coordinates are based on the UCSC Genome Browser Mouse July 2007 (NCBI37/mm9) Assembly.

### Statistical analyses

Consistent with previous studies examining strain differences in behavior, in our preliminary analyses we found significant effects of strain in all behavioral tests conducted, with B6 mice exhibiting increased time spent in the center area of the open-field (*p* < 0.001), longer latencies to enter the inner area (*p* < 0.001), increased average sucrose consumption (*p* < 0.05), and a decreased likelihood of engaging in aggressive behavior (*p* < 0.05) compared to Balb/c mice. Thus, for analyses of rearing condition effects, we analyzed each strain separately. Open-field data (time spent in the center area, total activity) were analyzed using 2-way ANOVA, with sex and rearing condition as independent variables and litter as a covariate. Latency data (time to enter the center area, social approach) were analyzed with Kaplan–Meier survival analysis. For sucrose consumption data, a χ^2^ test was conducted to determine group differences in likelihood of exhibiting sucrose preference (>75% sucrose consumption). Similarly, a χ^2^ test was conducted to determine group differences in likelihood of engaging in aggressive behavior (males only). For gene expression and DNA methylation analyses, we found significant strain by brain interactions and analyzed data from each strain and brain region using separate 2-way ANOVAs with sex and rearing condition as independent variables. For DNA methylation analyses, average CpG methylation levels across the multiple CpG sites assessed was used in the ANOVA.

## Conflict of Interest Statement

The authors declare that the research was conducted in the absence of any commercial or financial relationships that could be construed as a potential conflict of interest.
